# Correlated-Weighted Statistically Modeled Contourlet and Curvelet Coefficient Image-Based Breast Tumor Classification Using Deep Learning

**DOI:** 10.3390/diagnostics13010069

**Published:** 2022-12-26

**Authors:** Shahriar M. Kabir, Mohammed I. H. Bhuiyan

**Affiliations:** 1 Department of Electrical and Electronic Engineering, Green University of Bangladesh, Dhaka 1207, Bangladesh; 2 Department of Electrical and Electronic Engineering, Bangladesh University of Engineering and Technology, Dhaka 1000, Bangladesh

**Keywords:** convolutional neural network (CNN), machine learning, deep learning, breast cancer, contourlet, curvelet, B-mode ultrasound, Rician inverse Gaussian, parametric image

## Abstract

Deep learning-based automatic classification of breast tumors using parametric imaging techniques from ultrasound (US) B-mode images is still an exciting research area. The Rician inverse Gaussian (RiIG) distribution is currently emerging as an appropriate example of statistical modeling. This study presents a new approach of correlated-weighted contourlet-transformed RiIG (CWCtr-RiIG) and curvelet-transformed RiIG (CWCrv-RiIG) image-based deep convolutional neural network (CNN) architecture for breast tumor classification from B-mode ultrasound images. A comparative study with other statistical models, such as Nakagami and normal inverse Gaussian (NIG) distributions, is also experienced here. The weighted entitled here is for weighting the contourlet and curvelet sub-band coefficient images by correlation with their corresponding RiIG statistically modeled images. By taking into account three freely accessible datasets (Mendeley, UDIAT, and BUSI), it is demonstrated that the proposed approach can provide more than 98 percent accuracy, sensitivity, specificity, NPV, and PPV values using the CWCtr-RiIG images. On the same datasets, the suggested method offers superior classification performance to several other existing strategies.

## 1. Introduction

For both industrialized and developing nations, female breast cancer is a very pressing issue. According to a current report from the American Cancer Society’s Cancer Statistics Center, there are expected to be 1,918,030 new cases of breast cancer in 2022, while there will be only 609,360 cancer deaths in the country overall, with 290,560 of those cases (or roughly 48 percent) being breast cancer cases [1].

Among the other imaging modalities, such as mammography and MRIs, one of the most promising techniques is breast ultrasonography (US) imaging for classifying breast tumors. Numerous studies have been conducted and are continually being conducted to increase the precision of classifying benign from malignant breast tumors automatically. The depth-to-width ratio, the normalized radial gradient, and the autocorrelation feature were three computer-extracted characteristics that were combined in 2002 by K. Horsch et al. [2] to detect breast cancers in the depth of lesion region. A computer-aided diagnostic (CAD) system was introduced in 2007 by Wei-Chih Shen et al. [3] The classification outcomes are estimated by mean values and standard deviations (SDs) of a shape, orientation, margin, lesion border, echo pattern, and posterior acoustic properties such as geometrical features. The accuracy is stated to be 91.7%, but in that study, from the healthy breast tissue, the lesion site was segmented both manually and automatically. In big data analysis of US images, manual lesion boundary detection can occasionally be challenging. Multi-resolution transform domain-based US imaging approaches have recently shown encouraging results in automated breast tumor classification tasks. To produce non-redundant data sets and more possible transform domain features, Sharmin R. Ara et al. [4] proposed an EMD method in 2017. This method applies discrete wavelet transform (DWT) and then a wrapper-based subset selection process follows. According to their dataset, the accuracy is 98.01%. Traditional DWT provides only horizontal, vertical, and diagonal dimensions in addition to limited information about directions. In 2019, P. Acevedo et al. [5] categorized benign and malignant tumors using a K-means and gray-level concurrency matrix (GLCM) approach with a linear support vector machine (SVM). A comparison study of two domains for multi-resolution transforms, such as the wavelet and curvelet transform domains, for the categorization of breast tumors in digital mammography images was presented by Eltoukhy et al. [6].

Another multi-resolution transform domain name contourlet transform-based technique is depicted in [7]. Pyramidal decomposition levels rising in the contourlet transform are accompanied by an expansion of several directional decomposition levels, which has proved to yield additional directional information. It has also been demonstrated that it is a more accurate descriptor of arbitrary forms and contours than wavelet, curvelet, and dual-tree complex wavelet transforms, as there are various multi-resolution transform domains, etc. The use of mammography images for contourlet-based mass classification is documented in [8,9]. Low-energy X-ray radiation is used in mammography for routine examinations. The lack of specificity in mammography has the disadvantage of forcing many women to undergo needless breast biopsies. Approximately 65%–85% of unnecessary breast biopsies are determined to be benign cases. It causes unnecessary biopsies and increases the unexpected cost of mammographic screening, which is a burden for the patients both emotionally and physically [10]. Because of this, researchers are becoming increasingly more interested in relatively safe methods, such as elastography and ultrasonography. As a way to judge the elastic heterogeneity of breast tissue, [11] uses the multiscale and multidirectional contourlet transform to extract texture information from shear–wave elastography (SWE) images. The Fisher classifier was used to classify the data, and the contourlet-based texture features reported an accuracy of 92.5%, proving that they are more accurate than the traditional characteristics in separating benign from malignant breast tumors. The unique concept of radiomics with attribute bagging was initially introduced in [12]. It uses the contourlet transform on B-mode ultrasound (US), contrast-enhanced US (CEUS), and shear–wave elastography (SWE) images and reports accuracy of 67.57%, 75%, and 81.08%, respectively. 

Contrary to the contourlet transform, the DWT has poor directional selectivity in two dimensions and is unable to give a wide range of directions. However, the curvelet transform also has a variety of directions. Breast tumor classification using a local binary pattern, the curvelet transform-based feature extraction method, is presented in [13] and reported to have an accuracy of 94.17%. Breast dynamic contrast-enhanced (DCE-MRI), magnetic resonance imaging with dynamic contrast, which is in the investigation, is utilized [14] to classify breast masses into benign and malignant utilizing curvelet characteristics. This technique produced a good diagnostic accuracy of 96%. Eltoukhy et al. [15] presented a breast cancer diagnosis using a multiscale curvelet transform and reported an accuracy of 98.59%. Statistical features are used in tissue classification using curvelet transform, and 85.48% accuracy is gained [16]. The most discerning textural characteristics of interest zones include are utilized in mass classification purpose using curvelet transform, and an accuracy of 91.68% is achieved in [17]. Sheeja et al. [18] utilized breast thermography to detect abnormality using curvelet transform, and 90.91% accuracy is shown for classification purposes. In [19], a special set of curvelet coefficients are used as the features from different medical masses and achieved a satisfactory accuracy rate for different (10–90%) ratios of coefficients. Karthiga et al. [20] demonstrated an accuracy of 93.3% using 16 statistical, geometrical, and intensity criteria for the automated classification of input thermal pictures. 

Statistical modeling is another imaging technique where various features are extracted from the statistical models, such as Gaussian or Nakagami images obtained from the original B-mode images, which is called parametric imaging [21,22], and has found satisfactory results in breast tumor classification. These statistical techniques were developed primarily to quantitatively simulate the sound waves dispersing through tissue, which can offer a deeper understanding of the system and more accurate features. More so than spatial domain visual ultrasound pictures, false positive (FP) and false negative (FN) results can be described statistically. Nakagami modeling was employed by Ming-Chih Ho et al. [23] to investigate the detection of rat liver fibrosis, which may not be the same as classifying breast tumors, but it does offer some support for the value of parametric imaging. The use of deep CNN, as a potential technique for the automatic interpretation of various medical image types, is a recent development in this field, enabling the quick and accurate identification of various medical diseases. Deep neural networks enable the development of automated medical solutions that are very efficient and very accurate, particularly for the automated categorization of breast tumors [24]. This is in contrast to conventional incorporated engineering-based methods, which depend on the accuracy of the feature extraction techniques for their resilience. Shear–wave elastography data were subjected to CNN and morphological information extraction methods by Zhou et al. [25] for the categorization of breast tumors. CNN was used for breast tumor categorization by Zeimarani et al. [26]; however, they applied it directly to breast ultrasound images. A generative adversarial network (GAN) and CNN were successfully used by Singh et al. [27] to separate and categorize breast tumors from ultrasound images. Ramachandran et al. [28] achieved a decent outcome in a small online dataset using a straightforward neural network that is inexpensive and simple to use. According to Hou et al. [29], a gadget itself without using a cloud-based server, a CNN classifier, may be trained using a model of a pre-trained AI neural network. The research of Shin et al. [30] showed that a neural network with quicker R-CNN and ResNet-101 was possible. A technique for converting US to RGB and fine-tuning it through back-propagation was published by Byra et al. [31]. With multiple-scale kernels and skip connections, Qi et al. [32] demonstrated a unique deep CNN technique. Deep neural network approaches, however, do not consider statistical aspects or traits.

In this study, it is demonstrated that an extremely successful model is the Rician inverse Gaussian (RiIG) distribution, which comprises the statistics of the contourlet and curvelet coefficient images [33]. It has been demonstrated that the features derived by the CNN network from the RiIG statistically modeled (i.e., parametric) images, compared with features extracted from US B-mode images, provide a higher level of accuracy for breast tumor classification. Firstly, the contourlet and curvelet transform are applied to the US B-mode images to obtain contourlet and curvelet coefficient (C) images. The next step is to create contourlet or curvelet parametric (CP) images by substituting a pixel from the coefficient (C) image with the estimated RiIG parameter (δ), which is carried out over a local neighborhood of the corresponding pixels with the requested parameter taken into account at the neighborhood’s center. The parameter values that produce the CP image are, therefore, transformed (δ-mapped) from the pixel values. To enhance the precision of the statistical characteristics in classification, correlated-weighted contourlet (Ctr)- or curvelet (Crv)-transformed parametric (CWCtrP or CWCrvP) images are introduced. The contourlet or curvelet parametric (CP) images are correlated with their matching contourlet- or curvelet-transformed coefficient (C) images to create the CWCtrP or CWCrvP images. As a result of applying correlation with the relevant contourlet or curvelet coefficient (C) images, weights were assigned to each parameter of CP images; the term “correlated-weighted” is being utilized in this system. In this work, the CWCtrP and CWCrvP images are used to classify breast tumors in a deep CNN architecture. The proposed methods subject fully connected layers and a variety of machine learning classifiers, including the support vector machine (SVM), k-nearest neighbor (KNN), random forest, etc., to the features extracted from the proposed deep CNN’s global average pooling layer.

The features recovered from the database US B-mode images, parametric (P) images, contourlet-converted (C) images, contourlet parametric (CP) images, and weighted contourlet parametric (WCP) images, have previously been demonstrated to have the highest level of accuracy [34]. So, only the correlated-weighted version of the contourlet- and curvelet-transformed parametric (CWCtrP and CWCrvP) images are examined in the proposed method rather than P, C, and CP images. In this context, the CWCtrP and CWCrvP images consisting of six contourlet and curvelet sub-band concatenated coefficients are fed to the deep CNN network separately for a comparative study of the two multi-resolution transform domain’s image performance. The pre-trained networks cannot be used with our six-channel stack of contourlet and curvelet transform domain CWCtrP and CWCrvP coefficient images since they are made for one- or three-channel visual images with spatial dimensions. As a result, a custom-made deep CNN architecture is provided. On three publicly accessible US image databases for identifying breast tumors, the performance of the prior classifiers is evaluated and compared with the state-of-the-art techniques. 

The following list summarizes this work’s significant contributions:

- The appropriateness of the Rician inverse Gaussian (RiIG) distribution for statistical modeling of both contoured and curvelet-transformed breast ultrasound images is demonstrated in this study.
- To assess the feasibility of correlated-weighted contourlet- and curvelet-transformed parametric (CWCtrP and CWCrvP) images in classifying breast tumors employing three distinct publicly available datasets, a new investigation is conducted.
- A new correlated-weighted contourlet-transformed RiIG (CWCtr-RiIG) and curvelet-transformed RiIG (CWCrv-RiIG) image-based deep CNN architecture is proposed.

## 2. Materials and Methods

### 2.1. Datasets 

This study examined 996 clinical cases in 1193 US images; Database-I (Mendeley Dataset) provided 250 of these cases, Database-II (Dataset UDIAT) provided 163, and Database-III ((Dataset BUSI)) provided 647. The Database-I, which is available at (https://data.mendeley.com/datasets/wmy84gzngw/), accessed on 28 February 2020, includes work by Rodrigues et al. [35]. There are 250 US images in this collection, 100 of which are fibroadenoma examples (benign), and 150 of which are malignant cases. The images are saved in the *.bmp format. The Database-II contains 163 US images in *.png format and is available at (http://www2.docm.mmu.ac.uk/STAFF/m.yap/dataset.php), accessed on 21 April 2020 [36]. Radiologists in this database identified the lesion regions (i.e., tumor outlines) of the 163 clinical cases and recorded them in binary image format in distinct folders from the B-mode US images. The pathological results of these 163 lesions were classified into various groups, including fibroadenoma (FA), invasive ductal carcinoma (IDC), ductal carcinoma in situ (DCIS), papilloma (PAP), unknown (UNK), lymph node (LN), and lymphoma (LP). There are 110 benign instances and 53 malignant cases. Database-III, which is accessible at (https://scholar.cu.edu.eg/?q=afahmy/pages/dataset), accessed on 13 December 2020 [37], contains 780 US images in *.png format. There are 600 female patients with baseline breast ultrasound scans in this collection, which includes women between the ages of 25 and 75. Totaling 780 images, the dataset includes 437 benign, 210 malignant, and 133 normal samples. Only the benign and malignant examples (647 pictures out of 780) are included in this study for categorization purposes. The ground truth photos are shown alongside the original images. Table 1 lists the specifics of these three datasets. In general, deep neural networks demand a great deal of computing power. Because of how the augmentation was implemented, there were 1000 benign and 1000 malicious instances in each of the three databases. There were 2000 images per database, which comprised the 6000 total augmented images at that time. To equalize the number of benign and malignant cases, data augmentation was carried out primarily by expanding the sample numbers needed to train the neural network and eliminate the class disparity. As is typical of clinical scanner outputs, the images in these earlier databases had previously undergone pre-processing (such as edge enhancement, speckle reduction, compressed dynamic range, persistence, etc.). As a result, additional pre-processing steps for removing different sounds, artifacts, and anomalies are not required. The following sub-sections outline the essential processes for preparing the database images for the dual input CNN architecture.

#### 2.1.1. Contourlet Transform

**Figure 1:**
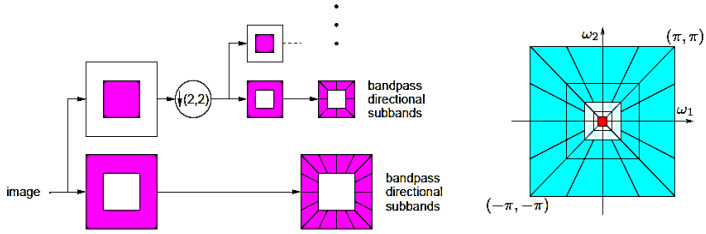
Filter bank for contourlets.

Since the standard discrete wavelet transform (DWT) domain includes only horizontal, vertical, and diagonal dimensions, it offers limited dimensional information. The contourlet transform, on the other hand, supports a wide range of arbitrary forms and contours that are not restricted to three dimensions. The normalized B-mode images are transformed using the contourlet transform to use a filter bank to separate the directed and multiscale decompositions [7], as illustrated in Figure 1. 

#### 2.1.2. Curvelet Transform

**Figure 2:**
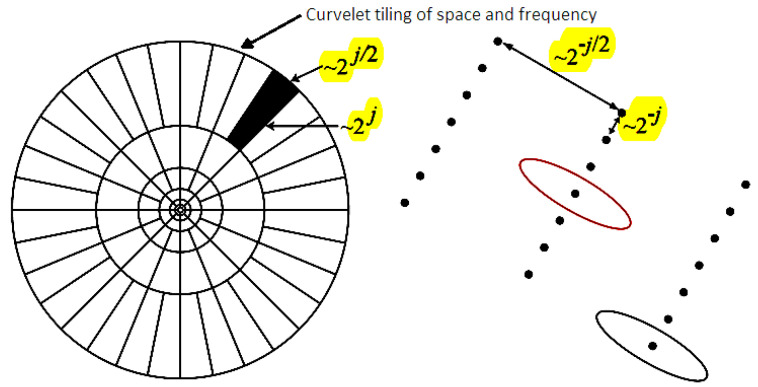
Curvelet transform provides an optimal sparse representation of objects with edges and contours.

The best sparse representation of objects with edges and contours is provided by the curvelet transform. In contrast to the isotropic components of wavelets, the needle-shaped elements of the curvelet transform have extremely high directional sensitivity and anisotropy. Later, the second-generation curvelet transform was demonstrated to be a very effective tool for a variety of applications, including partial differential equations, seismic data exploration, image processing, and fluid dynamics (PDEs). Periodization was used to treat image borders in earlier iterations of the transform previous version. The data have been properly arranged in this case, and the discrete cosine domain will be tiled instead of the discrete Fourier domain, which is a significant change. A discrete filter bank structure, called smooth images with piecewise smooth contours, can be handled via contourlets. Structures resembling curvelets in the continuous domain can be coupled to this discrete transform. As a result, the contourlet transform can be considered a discrete version of a certain curvelet transform. Figure 2 illustrates how curvelet constructions connect to a polar coordinate-based partition of the 2-D frequency plane [38] and call for a rotational operation. 

**Figure 3:**
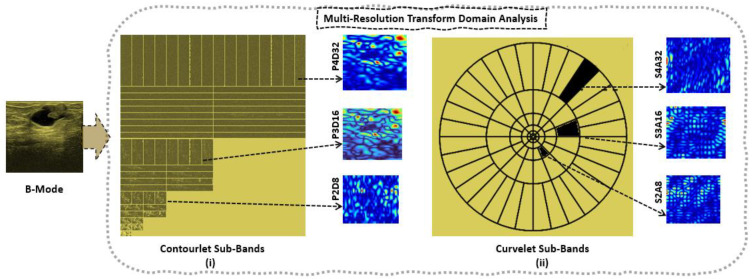
B-Mode to multi-resolution transform domain analysis: (**i**) Contourlet sub-band coefficient images at pyramidal decomposition level-2 directional decomposition level-8 (P2D8), as well as P3D16, and P4D32; (**ii**) Curvelet sub-band coefficient images at decomposition levels: scale-2 angle-8 (S2A8), as well as S3A16 and S4A32.

Figure 3 compares the effectiveness of the contourlet transform and curvelet transform in terms of improved descriptors of contour segments. With increasing decomposition levels for both the contourlet and curvelet transform, it can be noticed that the contour detection grows smoother as the range of the 32 dimensions increases. The literature claims that the contourlet transform can also offer a more accurate description, random form definitions, contours, and additional directional information [7,38]. The pyramidal decomposition levels rise along with an increase in the directional sub-bands, and there are numerous variable orientations seen in the directional decomposition levels. A crucial component of contourlets, the directional filter bank, has a practical tree structure where aliasing is permitted to occur and will be removed by correctly designed filters. Because of this, the primary distinction between contourlets and curvelets is that the former is explicitly specified on discrete rectangular grids, which are easier to digitize. Unfortunately, contourlet functions exhibit more oscillations along the needle-like elements than curvelets and exhibit less well-defined directional geometry/features. This results in artifacts in denoising and compression.

#### 2.1.3. Contourlet and Curvelet Parametric (CP) Image

Rician inverse Gaussian (RiIG) image

**Figure 4:**
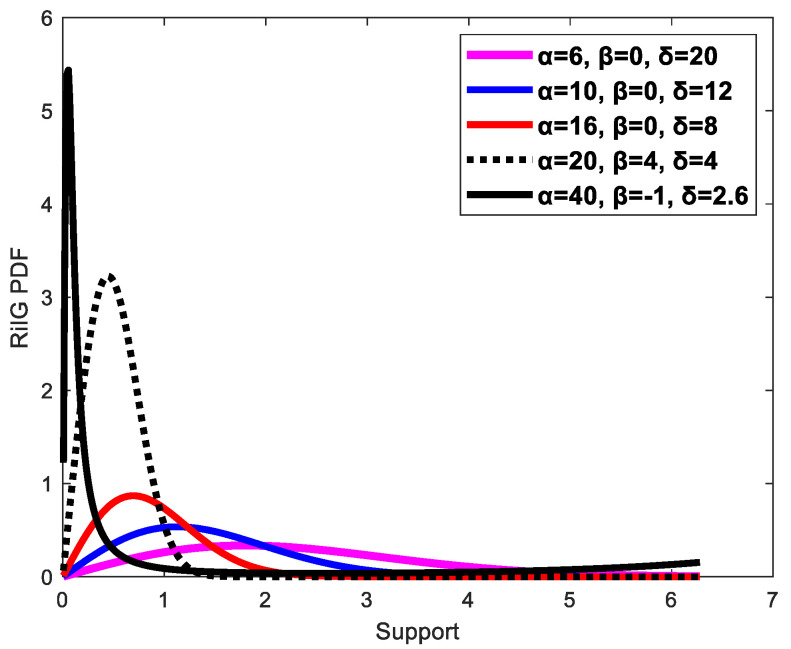
Various examples of the RiIG model’s pdfs with different *α*, *β*, and *δ* values.

Eltoft et al. [33] introduced the RiIG distribution, which is a mixture of Rician and inverse Gaussian distributions and is expressed as:

(1) $$\;P_{RiIG}\left(r\right)=\sqrt{\frac{2}{\pi }\alpha^{\frac{3}{2}}}\delta \;exp\left(\delta \gamma \right)\times \frac{r}{{\left(\delta^{2}+r^{2}\right)}^{\frac{3}{4}}}K_{\frac{3}{2}}\left(\alpha \sqrt{\delta^{2}+r^{2}}\;\right)I_{0}\left(\beta r\right)$$

where *α* and *β* affect the distribution’s steepness and skewness respectively; *β* < 0 indicates a distribution that is skewed to the left, and *β* > 0 indicates one that is slanted to the right, whereas *δ* is the dispersion parameter. The value of γ can be calculated as $\gamma =\sqrt{\alpha^{2}-\beta^{2}}$. Figure 4 displays a selection of RiIG pdf realizations for different parameter values.

**Figure 5:**
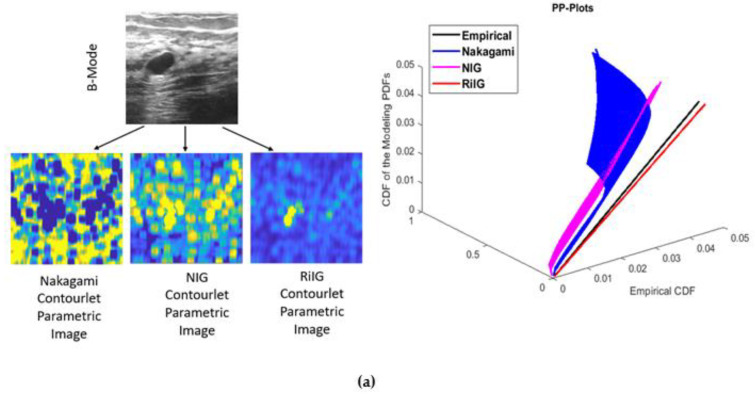

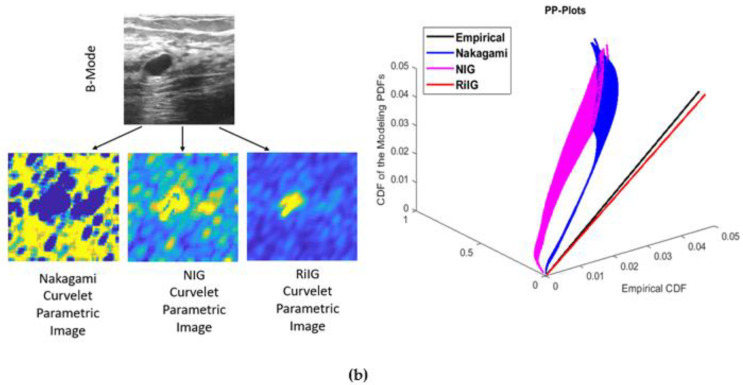
Comparing Nakagami, NIG, and RiIG statistical modeling with the aim of classifying images with (**a**) contourlet parametric (CP) images and (**b**) curvelet parametric (CP) images using percentile probability plots (pp-plots) that show empirical, Nakagami, NIG, and RiIG cumulative density functions (CDFs). It can be seen from both pp-plots that the RiIG CDF, as opposed to the Nakagami and NIG CDFs, closely tracks the empirical CDF. Additionally, it shows that for parametric modeling of breast ultrasound pictures, the RiIG distribution is more appropriate.

It is seen that, with an increase in *α* and *β*, the distribution becomes steeper and skews to the right, respectively. On the other hand, as *β* decreases, it skews to the left. In addition, as δ  is increased, the distribution becomes more dispersed. The RiIG parameter (*δ*) map is created by processing the contourlet and curvelet coefficient images via a square sliding window, which results in the contourlet and curvelet parametric (CP) image. For the Nakagami image, parameter (m) mapping and NIG image as well as parameter (α) mapping are considered. This procedure is shown in [22], where the author constructed a Nakagami parametric image using this procedure while computing the image parameters for each image. It should be mentioned that while we created the images in the domains of the contourlet and curvelet transforms, the literature [22,39,40] obtained the parametric images in the spatial domain. According to earlier research, the best sliding window for producing sides that are the parametric image is a square and has a pulse duration that is three times that of the incident ultrasound. In this study, each local RiIG parameter (*δ*) was examined utilizing the contourlet and curvelet sub-band efficient images with a sliding window of 13 × 13 pixels. The size of the sliding window that is being used should be larger than the speckle and be able to discern different local structure differences in malignancies. The new pixel added to the window’s center at each point when the window was moved across the entirety of the contourlet and curvelet sub-band efficient pictures in steps of 1 pixel was designated as the local RiIG parameter (*δ*). The map of RiIG parameter values produced by this technique is known as the RiIG parametric image. With the relevant figures and a percentile probability plot (pp-plot), the RiIG statistical model is already proven to be preferable to the Nakagami statistical model [34]. In this study, the appropriateness of RiIG statistical modeling over the Nakagami and normal inverse Gaussian (NIG) statistical models is shown in Figure 5 by contourlet and curvelet parametric images and percentile probability plot (pp-plot).

#### 2.1.4. Correlated-Weighted Contourlet- or Curvelet-Transformed RiIG (CWCtr-RiIG or CWCrv-RiIG) Image

**Figure 6:**
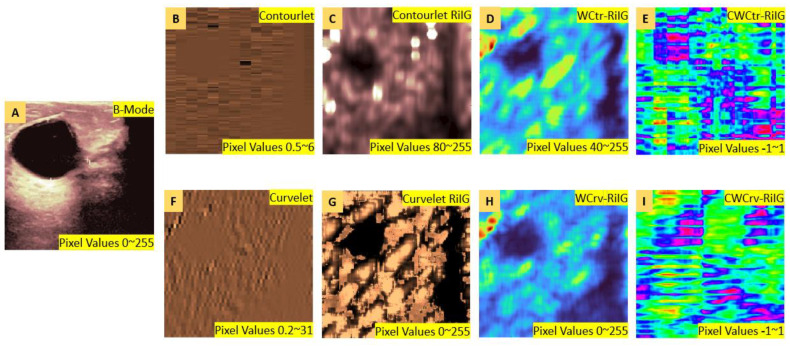
Example of transformation from B-Mode to CWCtr-RiIG and CWCrv-RiIG images at contourlet decomposition level-P4D32 and curvelet decomposition level-S4A32. (**A**) B-mode image, (**B**) Contourlet coefficient image, (**C**) RiIG modeled contourlet coefficient image, (**D**) RiIG modeled weighted contourlet coefficient (WCtr-RiIG) image, (**E**) RiIG modeled correlated-weighted contourlet coefficient (CWCtr-RiIG) image, (**F**) Curvelet coefficient image, (**G**) RiIG modeled curvelet coefficient image, (**H**) RiIG modeled weighted curvelet coefficient (WCrv-RiIG) image, and (**I**) RiIG modeled correlated-weighted curvelet coefficient (CWCrv-RiIG) image.

**Figure 7:**
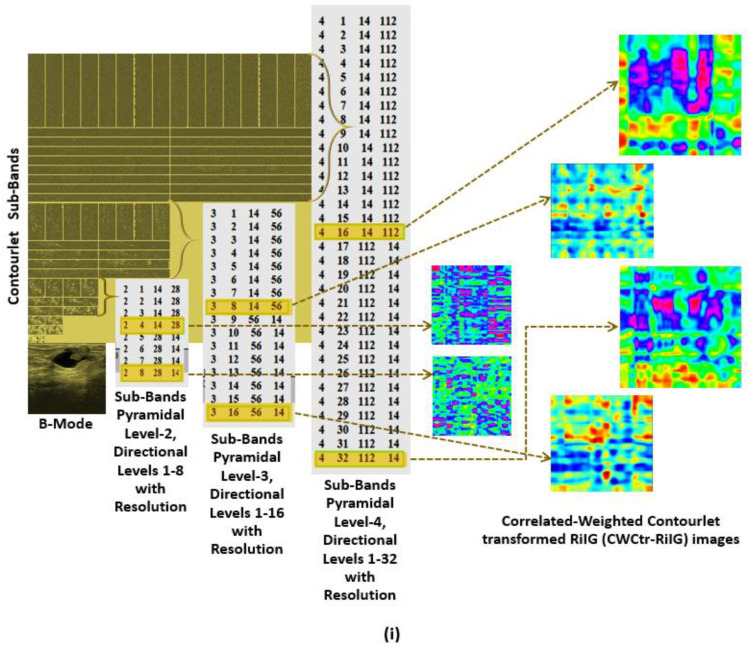

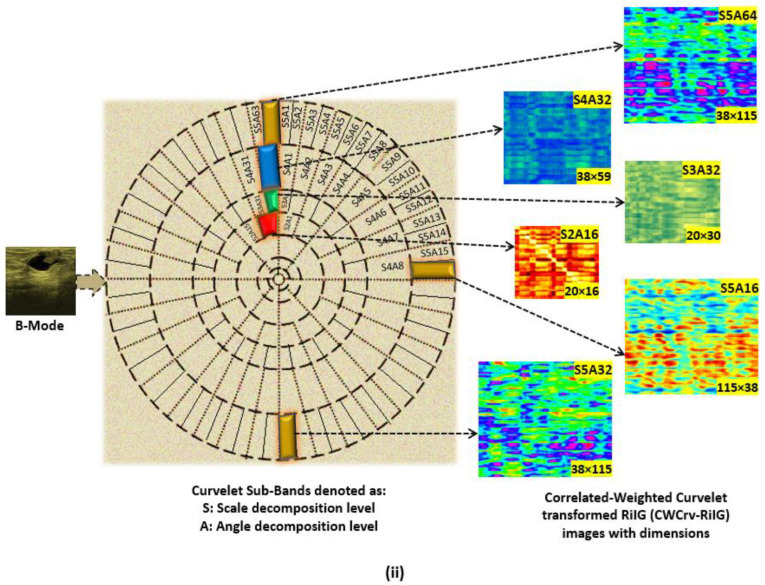
RiIG modeled correlated-weighted images: (**i**) CWCtr-RiIG images at pyramidal decomposition level-2, directional decomposition level-4 (P2D4), as well as P2D8, P3D8, P3D16, P4D16, and P4D32; (**ii**) CWCrv-RiIG images at decomposition levels, scale-2 angle-16 (S2A16), as well as S3A32, S4A32, S5A16, S5A32, and S5A64.

The CP images are linked with the appropriate contourlet and curvelet sub-band coefficient images to produce the CWCtr-RiIG and CWCrv-RiIG images. By executing correlation operations with the corresponding contourlet and curvelet sub-bands, the CP images’ parameter values are all weighted. The term “correlated-weighted contourlet- or curvelet-transformed RiIG” might be used to describe these images. Figure 6 displays the transformation from B-Mode image to correlated-weighted parametric imaging at contourlet decomposition level P4D32 and curvelet decomposition level S4A32 with corresponding image pixel value ranges. The transformation is progressing as at first the B-mode is transformed to a contourlet or curvelet transform coefficient image, then modeled by RiIG to obtain a contourlet or curvelet RiIG image. For comparison purposes, WCP [34] images (i.e., contourlet or curvelet coefficient are weighted by multiplication with their corresponding RiIG image to obtain WCtr-RiIG and WCrv-RiIG images) are also simulated. At last, the CWCtr-RiIG and CWCrv-RiIG images are simulated, except weighted by correlation rather than multiplication. To reduce the computational time for constructing CWCtr-RiIG and CWCrv-RiIG images, six sub-bands from the contourlet transform’s pyramidal decomposition at levels 2, 3, and 4 and the curvelet transforms decomposition at scales 2, 3, 4, and 5 are carefully selected as being the most ideal for feature extraction, where in contourlet, those pyramidal levels contain 8, 16, and 32 directional sub-bands, and in curvelet, those scale levels contain 16, 32, 32, and 64 angle sub-bands, respectively. In this study, contourlet directional sub-bands in each pyramidal level and curvelet angle sub-bands in each scale with larger sizes are taken into consideration because they predicted the best results than the other sub-bands. Therefore, the chosen sub-bands for contourlet analysis are pyramidal level-2 directional level-4 (P2D4), as well as P2D8, P3D8, P3D16, P4D16, and P4D32, which are shown in Figure 7i. For the curvelet domain, the chosen sub-bands are scale-2 angle-16 (S2A16), as well as S3A32, S4A32, S5A16, S5A32, and S5A64, which are shown in Figure 7ii. As previously stated, the primary rationale for choosing these sub-bands is that they offer the maximum resolution for the images, which is crucial for the classification process.

### 2.2. Proposed Classification Schemes

**Figure 8:**
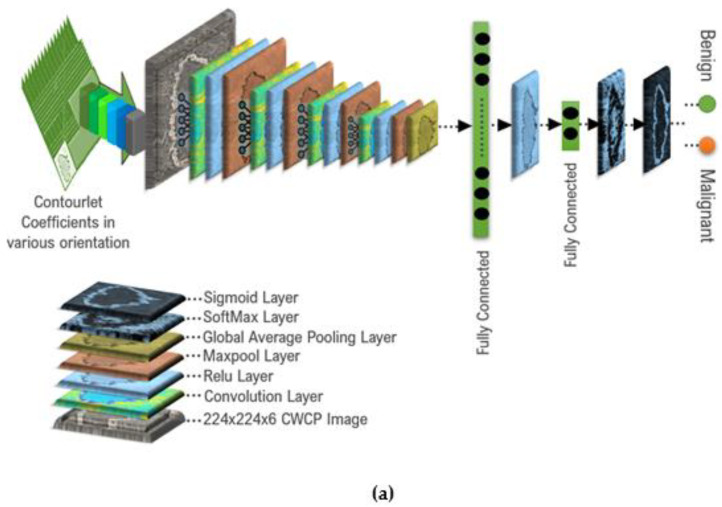

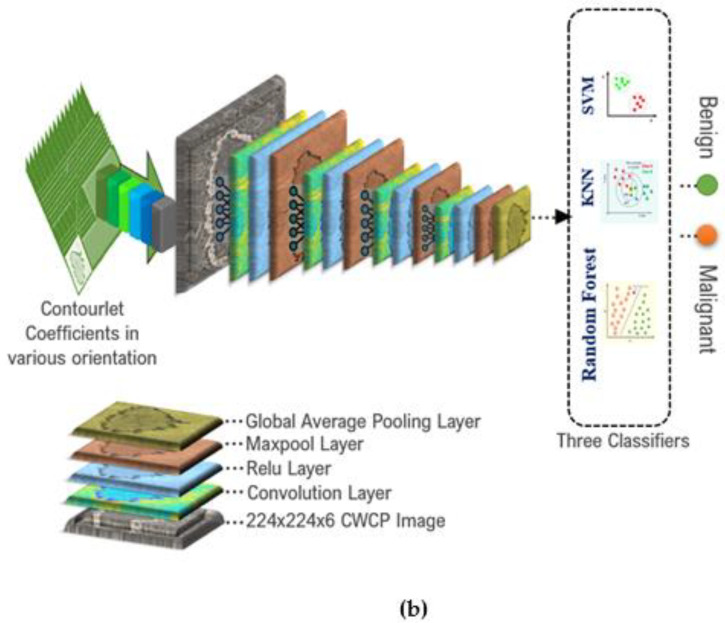
The proposed classification schemes (**a**) a deep CNN-based approach and (**b**) a machine learning classifier-based approach.

The proposed correlated-weighted statistically modeled contourlet and curvelet coefficient image-based classification schemes are illustrated in Figure 8. To inspect the performance of the deep CNN fully connected classifier and three machine learning classifiers, namely SVM, KNN, and random forest, are considered in this study. All of the classifiers used in this study were created in MATLAB (default parameters and the toolbox). In both, the deep CNN-based classification scheme and machine learning classification scheme, the correlated-weighted contourlet-transformed RiIG (CWCtr-RiIG) with 224 × 224 × 6 dimension stack images are applied as the input. For training, neural networks frequently need many more samples than the 250 images from Database-I, 163 images from Database-II, and 647 images from Database-III. To create three huge databases with a combined total of 6000 images, by augmentation, the sample count was raised to 2000 for each of the three databases, with an equal proportion of malignant and benign instances. Each B-mode image has six sub-bands, increasing the total number of images to 6000 × 6 = 36,000 contourlet coefficient and 36,000 curvelet coefficient images. As any form of scaling or rotation would likewise eliminate the features dependent on size or orientation, on the base images, only the translational augmentation of 1 to 11 pixels in both directions is carried out. The overall process is implemented by “imageDataAugmenter” MATLAB function. Figure 7 makes it clear that the images produced by various curvelet and contourlet sub-band coefficients all have distinct sizes. All of the images are enlarged to 224 × 224 because a CNN requires all of the images to be the same size. Then, 6000 stack images are produced by stacking the appropriate six sub-band images that are 224 × 224 × 6 in size. The CNN network employed in this work is inspired by the custom CNN network provided in [34] and has 375,500 parameters with weighted contourlet parametric (WCP) images. The differences between that scheme and our proposed scheme are that the proposed deep CNN architecture has 316,400 parameters employing 224 × 224 × 6 stack CWCtr-RiIG or CWCrv-RiIG images as input in two different multi-resolution transform domains, such as contourlet and curvelet transform domains, respectively. In the deep CNN-based approach, an activation function is generated by combining SoftMax and sigmoid functions, and in the machine learning-based approach, features are taken out of the deepest CNN outer layer (the Global Average Pooling layer), and those are applied to three different machine learning classifiers such as SVM, KNN and Random Forest. The proposed network is also tested with WCP images which are constructed by multiplication [34]. The suitability of CWCtr-RiIG and CWCrv-RiIG images over the WCP image is shown in Figure 6 and Table 2. It is observed that the CWCtr-RiIG and CWCrv-RiIG images have less training time in the same proposed deep CNN network than the WCP image. Moreover, the WCP image has pixel values from 40 to 255 in. 

Regarding contourlet transform domain and curvelet transform domain, when applied to the CNN network, those 0-to-255-pixel value images are normalized and variations will be less because the pixel values such as 255 and higher than 200 will be converted to pixel value 1, thus having fewer variations. On the other hand, the CWCtr-RiIG and CWCrv-RiIG image pixel values are −1 to 1, having more variations when normalized in a deep CNN network, which will be an impact on feature extraction. Table 3 shows the suggested deep CNN network configuration’s architecture. To ensure that the testing and training samples are completely separate, a ratio of 90% to 10% is employed for training, with 10% of the unaugment database photos and their matching augmented images randomly chosen for testing, and the remaining 90% is used for training. The accuracy can be significantly biased and higher than the genuine test if the test data and training data coincide. The hyper-parameters of the neural network are chosen using a statistic called the average validation accuracy together with a tenfold cross-validation scheme and an exhaustive grid search approach. The batch size and learning rate for this network are 60 and 0.01, respectively, along with the Adam optimization algorithm [41]. Through 4000 cycles, the CNN network is used to apply the training data. Utilizing accuracy, sensitivity, specificity, PPV, NPV, and other performance indicators, the proposed technique’s performance is evaluated. Once the TP, TN, FP, and FN signals have been measured, the confusion matrices have been constructed. True positive (TP) signals denote a malignant tumor and true negative (TN) signals, a benign tumor. Section 3 of the report discusses the conclusions. 

## 3. Results

The suggested classification schemes evaluate the classification performance on correlated-weighted parametric versions of contoured and curvelet-transformed images for both schemes. The findings are displayed in Table 4, where it is clear that the use of statistical modeling on the contourlet and curvelet transforms increases classification accuracy. Here, Database-I, -II, and -III all have the highest levels of accuracies by SVM classifier of 97.05%, 97.35%, and 98%; by KNN classifier, 97.85%, 98.05%, and 98.25%; by random forest classifier, 98.15%, 98.40%, and 98.85%; and by deep CNN classifier 98.25%, 98.45%, and 98.95%, respectively. RiIG modeling’s appropriateness on B-mode images instead of Nakagami, Gaussian, and normal inverse Gaussian (NIG) statistical models were already depicted in a few works in the earlier literature [33,34]. In this study, the accuracy is also compared with Nakagami and NIG statistically modeled as CWCrv-Nakagami, CWCtr-Nakagami, CWCrv-NIG, and CWCtr-NIG images along with the RiIG modeled correlated-weighted images, and it is observed that RiIG is highly suitable for correlated-weighted transform domain parametric images in breast tumor classification. From the results, it is seen that the deep CNN classifier has the best result in the classification performance. A new activation function is applied here combining SoftMax and sigmoid activation functions. SoftMax function provides the softened maximum probability in multiclass classification. The correlated-weighted contourlet- or curvelet-transformed RiIG images have pixel values −1 to 1. In a few cases, the maximum probability of two classes (i.e., benign and malignant) appaired the same. By adding the sigmoid activation function, which provides a hard decision (e.g., benign or malignant), discrimination in such classes would be possible. The SoftMax function is given by $\sigma {\left(\vec{z}\right)}_{i}=\frac{e^{z_{i}}}{\sum_{j=1}^{k}e^{z_{j}}}\;$, where $\vec{z}$ is the input vector, $e^{z_{i}}$ is a standard exponential function for the input vector, and $e^{z_{j}}$ is a standard exponential function for the output vector with the multi-class classifier, having k classes in total. For multiclass classification SoftMax, the activation function is a better choice. In this paper, by using only the SoftMax function, the accuracy, sensitivity (true positive rate), and specificity (true negative rate) are attained at 98.95%, 99.19%, and 98.71%, respectively, with an F1 score of 0.989. Another activation function having nonlinear boundary decision is the sigmoid function defined as $\sigma {\left(\vec{z}\right)}_{i}=\frac{1}{1+e^{-z_{i}}}$, where e is Euler’s number. Combining the SoftMax function with the sigmoid function a new activation function is generated, which can be defined as:

(2) $$\;\;\;\;\sigma {\left(\vec{z}\right)}_{i}=\frac{e^{z_{i}}}{\sum_{j=1}^{k}e^{z_{j}}}+\frac{1}{1+e^{-z_{i}}}\;\;\;\;\;\;\;$$

**Figure 9:**
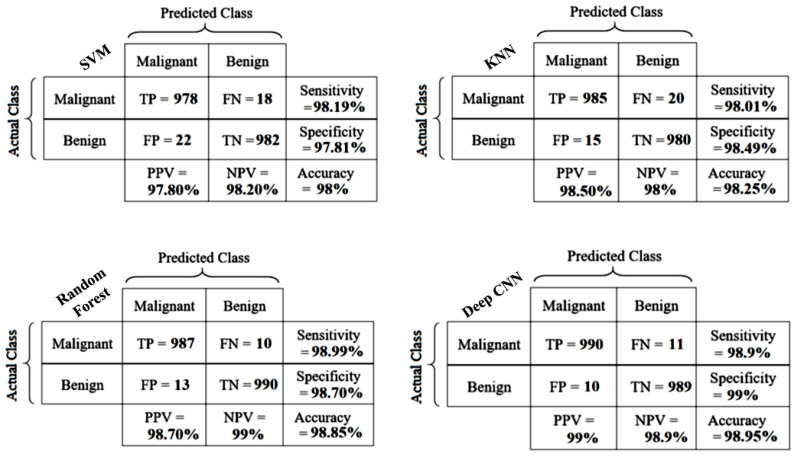
The confusion matrices according to the best performance achieved in Database-III.

Applying this combined activation function, the accuracy, sensitivity (true positive rate), and specificity (true negative rate) is attained at 98.95%, 98.9%, and 99%, respectively, with an F1 score of 0.99, which means that although the accuracy is not changed, the F1 score slightly increases. In the deep learning-based classification task, each value after a point with a high accuracy (such as 98%) is significant. Table 4 makes it clear that RiIG was more appropriate for the B-mode statistically modeled images, as it proved to be more effective than the Nakagami and NIG statistical models for all four classifiers in Database-I, -II, and -III. Additionally, the findings showed that a deep CNN-based classification scheme with a fully connected classifier provided better accuracy than other machine learning classifiers. In the SVM machine learning classifier-based approach, the highest accuracy is obtained in Database-III, where the accuracy, sensitivity, specificity, PPV, and NPV are 98%, 98.19%, 97.81%, 97.80%, and 98.20%, respectively. In the case of KNN, the highest performance is obtained in Database-III, where the accuracy, sensitivity, specificity, PPV, and NPV are 98.25%, 98.01%, 98.49%, 98.50%, and 98%, respectively. In the case of random forest, the best performance is also obtained in Database-III, where the accuracy, sensitivity, specificity, PPV, and NPV are 98.85%, 98.99%, 98.7%, 98.7%, and 99%, respectively. For the deep CNN fully connected classifier the overall best performance is obtained regarding the accuracy, sensitivity, specificity, PPV and NPV are 98.95%, 98.9%, 99%, 99%, and 98.9%, respectively. The suggested RiIG-based CWCtr-RiIG images are the best option for the categorization of breast tumors in both the deep CNN fully connected classifier-based approach and the machine learning classifier-based approach, as shown in Table 4. The confusion matrices in terms of best performance with Database-III, shown in Figure 9, display the suggested methods using the deep CNN, SVM, KNN, and random forest classifiers along with performance indices such as accuracy, sensitivity, and specificity as well as PPV and NPV, involving measuring malignant tumors as true positives (TP), benign tumors as true negatives (TN), false positives (FP), and false-negatives (FN), respectively. The greatest values of accuracy, sensitivity, specificity, PPV, and NPV for Database-III, utilizing both categorization systems, are seen to be greater than 98%.

## 4. Discussion

**Figure 10:**
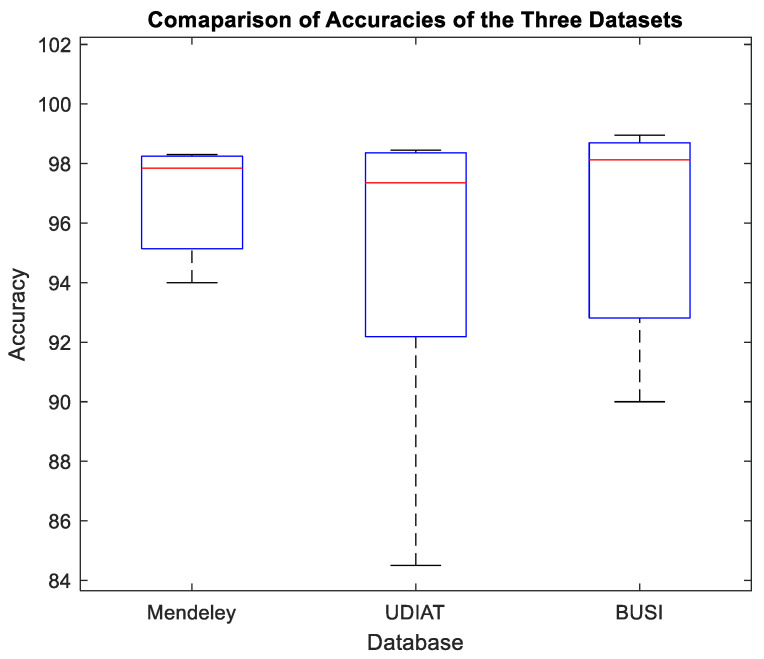
Performance indices comparison among the three databases from Table 5.

The best classification accuracy is demonstrated in the previous section using the deep CNN classifier with the RiIG-based CWCtr-RiIG pictures. Table 5 offers a comparison with comparable works. Using the same Database-I, P. Acevedo et al. [5] claimed a 94% accuracy, with a 0.942 F1 score, while Karthiga et al. [20] reported 94.5% accuracy, with a 0.945 F1 score. In light of this, the highest level of accuracy made possible by the proposed method employing the same Database-I is roughly 98.30% and has an F1 score of 0.983, which is noticeably better. Hou et al. [29] employed Database-II in a different study and reported a 94.8% accuracy rate. Combining the same Database-II with additional databases, Shin et al. [30] found an accuracy of 84.5%. According to Byra et al. [31], utilizing Database-II, their accuracy was 85.3%, and their F1 score was 0.765. With an F1 score of 0.942 with Database-II, Qi et al. [32] demonstrated an accuracy of 94.48%. On the other hand, the suggested approach employing Database-II offers the highest accuracy, 98.45%, and an F1 score of 0.985. With Database-I, Kabir et al. [34]’s accuracy was 98.25%, and their F1 score was 0.982; for Database-II, it was 98.35%, and their F1 score was 0.984; and for Database-III, it was 98.55%, and their F1 score was 0.986. The approach of Ka Wing Wan et al. [42] yields accuracy for Database-III of 90% using a random forest classifier and 91% using a CNN, with an F1 score of 0.83. The identical Database-III was used by Moon et al. [43], who reported 94.62% accuracy, with a 0.911 F1 score. The accuracy and F1 score of the suggested method, in comparison, are superior, with the greatest accuracy being roughly 98.95% and an F1 score of 0.99. Additionally, using the same validation strategy as in [42,43], the suggested dual input CWCtr-RiIG image-based deep CNN technique is deployed for classification on Database-III, with an 80% training to 20% testing ratio. With an F1 score of 0.98, this experiment’s accuracy, sensitivity, and specificity ratings are still better than those of [42,43]. The box plots in Figure 10 show a comparison of the accuracy of Table 5, and they also show that the proposed method performs consistently with other approaches. As mentioned earlier, the images in Database-I have undergone speckle reduction, compressed dynamic range, and persistence pre-processes. For Database-II and -III, the images have undergone edge enhancement, speckle reduction, and persistence only. Due to the heavily compressed dynamic range, the resultant accuracy using Database-I is lower than that of Database-II and -III. Moreover, if we combined the images of Database-II and -III, the classification accuracy attained 98.4%, while by combining the images of the three databases, the classification accuracy fell to 97.15%. Therefore, it seems that an automated edge enhancement process may further improve the performance in the case of Database-I. However, incorporating an edge enhancement technique will increase the complexity of the method. It is an interesting area of future exploration to develop a novel neural network architecture that can deliver a high degree of accuracy even with heavily compressed dynamic range images by additional pre-processing such as edge enhancement. 

## 5. Conclusions

In this paper, a novel approach to breast tumor classification is presented, employing RiIG statistically modeled correlated-weighted contourlet- and curvelet-transformed RiIG images in a deep CNN architecture. In the first approach, the RiIG statistically modeled CWCtr-RiIG and CWCrv-RiIG images are classified by deep CNN fully connected classifiers. In the second approach, the RiIG statistically modeled CWCtr-RiIG and CWCrv-RiIG images are classified by deep CNN-SVM, KNN, and random forest machine learning classifiers. It is demonstrated that a high level of accuracy can be attained by using the deep CNN fully connected classifier. Second, a brand new, specially created deep CNN architecture is suggested for classifying CWCtr-RiIG and CWCrv-RiIG images of breast tumors since it performs more accurately. Additionally, the suggested deep CNN design can use the loss function to provide extremely high levels of sensitivity, specificity, NPV, and PPV values, combining SoftMax and sigmoid activation functions. On benchmark publicly available datasets, both algorithms show superior classification performance to the state-of-the-art techniques. Additionally, the RiIG distribution is a distribution that is very well-suited for simulating the characteristics of the contourlet and curvelet transform coefficients of breast tumor images obtained in B-mode ultrasound. By applying the transformer model-based approach and including additional datasets, there is room for improvement.

## Figures and Tables

**Table 1:** Patient data overview.

**Database-I**			
**Tumor Type**	**Patients**	**Lesions**	**Method**
Fibroadenoma (Benign)	91	100	Biopsy
Malignant	142	150	Biopsy
**Database-II**			
**Tumor Type**	**Patients**	**Lesions**	**Method**
Cyst (Benign)	65	65	Biopsy
Fibroadenoma (Benign)	39	39	Biopsy
Invasive Ductal Carcinoma (Malignant)	40	40	Biopsy
Ductal Carcinoma in Situ (Malignant)	4	4	Biopsy
Papilloma (Benign)	3	3	Biopsy
Lymph Node (Benign)	3	3	Biopsy
Lymphoma (Malignant)	1	1	Biopsy
Unknown (Malignant)	8	8	Biopsy
**Database-III**			
**Tumor Type**	**Patients**	**Lesions**	**Method**
Benign	600	437	Reviewed by Special Radiologists
Malignant		210	
Normal		133	
Total patients = 996, lesions = 1193			

**Table 2:** Comparison with earlier methods in terms of computational complexity.

Multi-Resolution Transform Domain	Training Parameters Required	WCP or WCtr-RiIG Training Time Required	CWCtr-RiIG or CWCrv-RiIG Training Time
Contourlet [34]	375,500 parameters	3 min 10 s	1 min 50 s
Contourlet or Curvelet [Proposed Method]	316,400 parameters	2 min 30 s	1 min 10 s

**Table 3:** The envisioned architecture of the CNN network.

Layers	Input Size	Kernel Size	Stride	Output Size
Input	224 × 224 × 6			
Conv 1	224 × 224 × 6	6 × 6 × 64	2 × 2	112 × 112 × 64
Relu 1	112 × 112 × 64			112 × 112 × 64
Maxpool 1	112 × 112 × 64	2 × 2 × 64	1 × 1	112 × 112 × 64
Conv 2	112 × 112 × 64	5 × 5 × 64	2 × 2	56 × 56 × 46
Relu 2	56 × 56 × 46			56 × 56 × 46
Maxpool 2	56 × 56 × 46	2 × 2 × 46	1 × 1	56 × 56 × 46
Conv 3	56 × 56 × 46	4 × 4 × 46	2 × 2	28 × 28 × 32
Relu 3	28 × 28 × 32			28 × 28 × 32
Maxpool 3	28 × 28 × 32	2 × 2 × 32	1 × 1	28 × 28 × 32
Conv 4	28 × 28 × 32	3 × 3 × 32	1 × 1	28 × 28 × 16
Relu 4	28 × 28 × 16			28 × 28 × 16
Maxpool 4	28 × 28 × 16	2 × 2 × 16	1 × 1	28 × 28 × 16
Global Avg. Pool	28 × 28 × 16			28 × 28 × 16
Fully Connected	28 × 28 × 16			1 × 1 × 16
Relu 5	1 × 1 × 16			1 × 1 × 16
Fully Connected	1 × 1 × 16			1 × 1 × 2
SoftMax	1 × 1 × 2			1 × 1 × 2
Sigmoid	1 × 1 × 2			1 × 1 × 2
Class out	1 × 1 × 2			1 × 1 × 2

**Table 4:** The classification performances of different correlated-weighted curvelet (CWCrv) and contourlet (CWCtr) parametric images with Database-I, -II, and -III.

**Accuracy (%) with Database-I**						
**Classifier**	**CWCrv-Nakagami**	**CWCtr-Nakagami**	**CWCrv-NIG**	**CWCtr-NIG**	**CWCrv-RiIG**	**CWCtr-RiIG**
SVM	93.10	93.25	94.25	94.40	96.65	97.05
KNN	94.15	94.50	94.65	94.95	96.95	97.85
Random Forest	93.75	93.95	95.15	95.55	97.15	98.15
Deep CNN	94.40	94.65	95.45	95.60	97.90	98.25
**Accuracy (%) with Database-II**						
**Classifier**	**CWCrv-Nakagami**	**CWCtr-Nakagami**	**CWCrv-NIG**	**CWCtr-NIG**	**CWCrv-RiIG**	**CWCtr-RiIG**
SVM	93.35	94.00	93.80	94.75	96.15	97.35
KNN	93.70	94.65	94.85	95.15	96.75	98.05
Random Forest	93.95	94.45	94.95	95.65	97.05	98.40
Deep CNN	94.15	94.85	95.25	95.95	97.85	98.45
**Accuracy (%) with Database-III**						
**Classifier**	**CWCrv-Nakagami**	**CWCtr-Nakagami**	**CWCrv-NIG**	**CWCtr-NIG**	**CWCrv-RiIG**	**CWCtr-RiIG**
SVM	93.45	94.55	94.35	95.45	96.95	98.00
KNN	94.25	95.65	95.05	96.65	97.15	98.25
Random Forest	94.40	95.15	95.25	96.80	97.35	98.85
Deep CNN	94.95	95.90	96.05	97.15	98.05	98.95

**Table 5:** Using Database-I, -II, and -III, a comparison of chosen studies with the suggested classification scheme.

Author (Year)	Major Contribution	Database	Classifier	Performance (Accuracy in %)
P. Acevedo, (2019) [5]	Gray-level concurrency matrix (GLCM) algorithm	Database-I [35]	SVM	ACC: 94%, F1 Score: 0.942
R. Karthiga, (2021) [20]	Simple Convoluted Neural Network	Database-I [35]	CNN	ACC: 94.5%, SEN: 94.9%, SPEC: 94.1%, F1 Score: 0.945
D. Hou, (2020) [29]	Portable device-based CNN architecture	Database-II [36]	CNN	ACC: 94.8%
S.Y. Shin, (2019) [30]	Neural Network with R-CNN and ResNet-101	Database-II [36]	R-CNN	ACC: 84.5%
M. Byra, (2019) [31]	US-to-RGB Conversion and fine-tuning using back-propagation	Database-II [36]	VGG19 CNN	ACC: 85.3%, SEN: 79.6%, SPEC: 88%, F1 Score: 0.765
X. Qi, (2019) [32]	Deep CNN with multi-scale kernels and skip connections.	Database-II [36]	Deep CNN	ACC: 94.48%, SEN: 95.65%, SPEC: 93.88%, F1 Score: 0.942
S.M. Kabir, (2021) [34]	WCP Image-based Custom-made CNN architecture	Database-I [35]	Deep CNN	ACC: 98.25%, SEN: 98.49%, SPEC: 98.01%, F1 Score: 0.982
		Database-II [36]	Deep CNN	ACC: 98.35%, SEN: 98.11%, SPEC: 98.59%, F1 Score: 0.984
		Database-III [37]	Deep CNN	ACC: 98.55%, SEN: 98.21%, SPEC: 98.89%, F1 Score: 0.986
Ka Wing Wan, (2021) [42]	Automatic Machine Learning model (AutoML Vision)	Database-III [37]	CNN Random Forest	ACC: 91%, SEN: 82%, SPEC: 96%, F1 Score: 0.87 ACC: 90%, SEN: 71%, SPEC: 100%, F1 Score: 0.83
W.K. Moon, (2020) [43]	CNN including VGGNet, ResNet, and DenseNet.	Database-III [37]	Deep CNN	ACC: 94.62%, SEN: 92.31%, SPEC: 95.60%, F1 Score: 0.911
Proposed Method	CWCP Image-based CNN architecture with Fusion-oriented classification	Database-I [35]	SVM KNN Random Forest Deep CNN	ACC: 97.05%, SEN: 97.29%, SPEC: 96.82%, F1 Score: 0.97 ACC: 97.85%, SEN: 97.52%, SPEC: 98.19%, F1 Score: 0.979 ACC: 98.25%, SEN: 98.40%, SPEC: 98.11%, F1 Score: 0.982 ACC: 98.30%, SEN: 98.49%, SPEC: 98.11%, F1 Score: 0.983
		Database-II [36]	SVM KNN Random Forest Deep CNN	ACC: 97.35%, SEN: 98.50%, SPEC: 98.21%, F1 Score: 0.983 ACC: 98.05%, SEN: 98.29%, SPEC: 97.81%, F1 Score: 0.98 ACC: 98.40%, SEN: 98.59%, SPEC: 98.21%, F1 Score: 0.984 ACC: 98.45%, SEN: 98.21%, SPEC: 98.69%, F1 Score: 0.985
		Database-III [37]	SVM KNN Random Forest Deep CNN	ACC: 98%, SEN: 98.19%, SPEC: 97.81%, F1 Score: 0.98 ACC: 98.25%, SEN: 98.01%, SPEC: 98.49%, F1 Score: 0.983 ACC: 98.85%, SEN: 98.99%, SPEC: 98.70%, F1 Score: 0.988 ACC: 98.95%, SEN: 98.9%, SPEC: 99%, F1 Score: 0.99

## Data Availability

The data presented in this study are openly available in Dataset-I: (repository name, Mendeley Data) at (https://doi.org/10.17632/wmy84gzngw.1), accessed on 28 February 2020, reference number [35]. Dataset-II: (repository name, Department of Computing and Mathematics, Manchester Metropolitan University) at (https://doi.org/10.1109/JBHI.2017.2731873), accessed on 21 April 2020, reference number [36]. Dataset-III: (repository name, Cairo University Scholars) at (https://doi.org/10.1016/j.dib.2019.104863), accessed on 13 December 2020, reference number [37].
